# Effect of Methyl–β–Cyclodextrin and Trehalose on the Freeze–Drying and Spray–Drying of Sericin for Cosmetic Purposes

**DOI:** 10.3390/ph14030262

**Published:** 2021-03-15

**Authors:** Lorella Giovannelli, Andrea Milanesi, Elena Ugazio, Letizia Fracchia, Lorena Segale

**Affiliations:** 1 Department of Pharmaceutical Sciences, Università del Piemonte Orientale, 28100 Novara, Italy; andrea.milanesi@uniupo.it (A.M.); letizia.fracchia@uniupo.it (L.F.); lorena.segale@uniupo.it (L.S.); 2 Department of Drug Science and Technology, Università di Torino, 10125 Torino, Italy; elena.ugazio@unito.it

**Keywords:** cosmetic proteins, *Bombyx mori* cocoons, sericin, methyl–β–cyclodextrin, drying techniques

## Abstract

Sericin is a protein extracted from *Bombyx mori* silk cocoons. Over the last decade, this wastewater product of the textile industry has shown many interesting biological properties. This protein is widely used in the cosmetic and biomedical fields. In this study, sericin has been obtained via a High–Temperature High–Pressure degumming process, and was dried using the freeze–drying (fd) and spray–drying (sd) techniques. Proteins tend to collapse during drying, hence, sericin has been dried in the presence of two selected carrier agents: methyl–β–cyclodextrin and trehalose. The obtained powders have been analyzed using thermal investigation, microscopy (optical, SEM), and granulometric and spectroscopic analyses. Moreover, the percentage yield of the spray–drying process has been calculated. Both the agents were able to significantly improve the drying process, without altering the physico–chemical properties of the protein. In particular, the co–spray–drying of sericin with methyl–β–cyclodextrin and trehalose gave good process yields and furnished a powder with low moisture content and handling properties that are better than those of the other studied dried products. These characteristics seem to be appropriate and fruitful for the manufacturing of cosmetic raw materials.

## 1. Introduction

Proteins are considered to be suitable ingredients in the biomedical and cosmetic fields and are used in a variety of skin–care formulations. Thanks to their ability to bind water, proteins are characterized by film–forming properties that provide a smooth appearance and a feeling of softness upon the skin [1,2,3].

Proteins usually undergo drying via either freeze–drying (lyophilization) or spray–drying techniques, which are processes extensively employed to remove water from pharmaceuticals, biopharmaceuticals and foods, leading to an increase in their stability and shelf life. Both rhGH, which is the recombinant human growth hormone that is used to treat hypopituitary dwarfism, and insulin for inhalation have been converted into powder by lyophilization [4]. Spray–drying has been used to process immunoglobulin G (IgG) [5] and cosmetic proteins, such as hydrolyzed collagen [6].

During the removal of water, the complex protein structure is prone to collapse, causing denaturation. To preserve the original structure and therefore the properties of these molecules, it is necessary to avoid structural changes during drying. Carrier agents such as trehalose and sucrose allow the protein to be maintained by replacing the hydrogen bonds throughout spray–drying [7]. This “water replacement” theory gains even more importance in the freeze–drying process, in which lyoprotectants, such as polyols, mono–, di– and polysaccharides, are frequently used as functional process agents, due to their hydroxyl groups. Besides this theory, other hypotheses such as vitrification and water entrapment have been proposed to explain the stabilizing and preserving effect of those excipients. The vitrification theory states that during freeze–drying the protein is immobilized in a rigid, glassy sugar matrix and therefore the degradation drastically slows down, as the protein is unable to unfold thanks to its reduced molecular mobility. On the other hand, the water entrapment is based on the concept that excipients are able to form a cage around the protein, entrapping water molecules. All these theories result in stabilizing and preserving the structure of the protein [8,9,10,11,12,13]. Moreover, some of these substances, such as trehalose, perform the additional function of moisturizers in skin and hair–care formulations [14].

The freeze–drying and spray–drying techniques have also been used to dry sericin, a globular glycoprotein characterized by low stability during storage in the liquid state [8,15]. Sericin is a natural hydrophilic and gelatinous protein produced by the silkworm *Bombyx mori*. Its main role is binding the two fibroin filaments together, granting structural integrity to the cocoon. In this way, sericin can effectively protect the silkworm from the external environment during its pupal stage [16]. Sericin’s molecular weight varies from 10–20 kDa to 310–400 kDa; it consists of three different polypeptides (sericin A, B and C), which are distinguished by their position in the cocoon shell (from the outermost to the innermost layer, respectively) and their composition and solubility in hot water. Upon cooling, sericin shows sol–gel properties due to its conversion from the random coil to the β–sheet form [17].

Fibroin and sericin are the two main components that form raw silk fibers. In the textile industry, the fibroin fibers are extracted by detaching sericin via degumming procedures [16,18]. The removed sericin has been considered a wastewater product for many years [19]. In recent decades, sustainability and the circular–economy concept have acquired an increasingly important role in manufacturing processes and consumer culture [20]. A great deal of effort has been made to recover this protein, and numerous studies have demonstrated the intrinsic biological properties of sericin [21,22,23]. Its biocompatibility, biodegradability and the absence of skin–sensitizing or skin–irritating potential have meant that sericin is used in the pharmaceutical, cosmetic and food fields. Indeed, numerous studies have demonstrated its antioxidant, anti–aging, photoprotective, antibacterial, antiproliferative and immunomodulant properties [24,25,26,27]. In cosmetics, sericin is a high–value component in a number of skin–care products and make–up formulations, such as mascara and nail cosmetics, in association with silk fibroin. Moreover, sericin is present in foundation creams and eyeliners as a coating agent for talc, mica, titanium dioxide, iron oxide and nylon. This protein has also been included in sunscreen products containing triazines and cinnamic esters as UV filters, leading to the enhancement of the light–screening effect [28,29]. Furthermore, silk sericin resembles the natural moisturizing factor (NMF) [30] thanks to the moisture retention capacity of its hydroxyl groups. Sericin–containing products can thus be used to prevent trans–epidermal water loss (TEWL) and increase skin hydration [25]. Sericin has also been shown to provide an increase in skin elasticity, anti–wrinkle and anti–aging effects in creams and ointments [28].

Sericin shows strong affinity towards keratin, and it is therefore particularly suitable for hair–care products [31]. In fact, sericin hydrolysates are present in many hair–conditioning and straightening formulations. The proteins natural film–forming ability means that sericin can protect hair against damage, protein loss, roughness, dryness and swelling, as well as improving fiber flexibility [30].

In addition to polyols and linear carbohydrates, cyclodextrins (CDs) are becoming increasingly established as a new class of thermal stabilizers of liquid–protein formulations thanks to their anti–aggregation activity [32]. CDs are macrocyclic oligosaccharides that are composed of a different number of glycosidic units: 6, 7 and 8, respectively named α, β and γ [33,34]. Their main characterizing feature is their ability to form water–soluble complexes with lipophilic molecules that can be completely or partially included in CD cavities, which are a hydrophobic environment. The size of the CD cavity is particularly relevant for their complexation ability, indeed α–CDs are principally used to host aliphatic chains, whereas β–CDs have been demonstrated to effectively accommodate aromatic rings. These characteristics allow some amino acids, such as Phe, Tyr, His and Trp, to be hosted [35,36]. In particular, β–CD derivatives are generally singled out for their aggregation inhibitory properties towards protein molecules. Branched β–CDs and dimethyl–β–CD are able to inhibit the thermally and chemically induced aggregation of egg lysozyme and fibroblast growth factor [37]. Hydroxypropyl–β–CD can stop the interfacial aggregation of rhGH [38], while sulfobutylether–β–CD interacts both through inclusion processes and ionic interactions, leading to different aggregation rates with various kinds of proteins [32]. Although the mechanism is not yet fully understood and several concomitant factors can take place, CDs have been successfully utilized to improve protein drying. In fact, it has been observed that methylated–β–CDs and hydroxypropylated derivative CDs are effective in preserving the enzymatic activity of freeze–dried lactate dehydrogenase, and in protecting, with a surfactant–like effect, rhGH from denaturation at the air–water interface throughout spray–drying [32,39].

There are currently many known uses of CDs in the pharmaceutical, food and cosmetic fields [40,41,42]. In the cosmetic field, CDs are employed to protect guest molecules against light and oxidation, as is the case of kojic acid, which decomposes when exposed to light or heat; its inclusion complexes with CDs are also able to enhance the whitening action of kojic acid on skin [43]. CDs are used in perfumes and deodorant formulations to avoid the loss of the very volatile fragrance compounds by evaporation. Moreover, CDs are able to ensure long–lasting product effects via slow molecule release [44]. Some commercial formulations, such as creams with keratolytic activity, contain CDs as solubilizers of components such as salicylic acid. In other products, the solubilization activity of CDs is used to remove substances from the skin; this is the case in some make–up removers. Moreover, the use of methylated–β–CD has been proposed as a means to solubilize the aromatic substances present in hair lotions instead of ethyl alcohol [45].

In this work, a randomly methylated–β–CD and trehalose have been used as carriers to obtain sericin powders, via freeze–drying and spray–drying, from liquid protein dispersions that are products of the degumming of *Bombyx mori* cocoons. The efficacy of these carriers in the drying processes has been evaluated by comparing the physico–chemical and technological properties of the sericin powders that were obtained with and without methylated–β–CD and trehalose.

## 2. Results and Discussion

### 2.1. Characterization of Sericin Dispersions

While there is a variety of degumming techniques employed in manufacturing, the high–temperature and high–pressure process allows proteins that are valuable for other purposes to be recovered. In fact, the purification of sericin from the chemicals involved in other extraction processes is difficult [46]. However, the selected degumming method only entails physical treatment, and the recovery of sericin after the degumming procedure is therefore quite easy. Moreover, the sericin obtained via high–temperature and high–pressure has been shown to have the least toxic effect on the mouse fibroblast cell line L929 [47], and the technique is considered to be the best way to preserve sericin for biological applications. It is also favored because of its high values of extraction, which are associated with the highest molecular weight distribution [48].

All the above reasons contributed to the selection of the process for the degumming of sericin from *Bombyx mori* cocoons, at a ratio of 40 g purified water/g cocoon, in this work. Firstly, an aliquot of the obtained sericin dispersion was lyophilized to determine the extraction yield achieved during the degumming process. The results indicate that the sericin protein was efficiently removed from the cocoons; the protein concentration in the dispersion was 0.7 ± 0.1% *w*/*w*, which corresponds to an extraction efficiency over the total cocoon mass of 27.0 ± 1.6% (*n* = 3). This outcome confirms that the extraction of sericin was almost complete; as reported in the literature, the protein content is between 20% and 30% in a cocoon [16,49].

Subsequently, the sericin dispersions were dried via either lyophilization or spray–drying. Before the spray–drying process, the viscosity of the degummed dispersion, with or without the M and T, was determined at 25 °C. All of the analyzed dispersions were found to be below 300 mPa⋅s, and, therefore, within the processability threshold of viscosity for the spray dryer [50].

### 2.2. Characterization of Sericin Powders

#### 2.2.1. Visual Inspection and Morphology

The sericin powder obtained from the freeze–drying process (fd_S) was fluffy and voluminous, white in color. Moreover, it was characterized by poor handling due to electrostatic behavior and, probably, to the protein aggregation. In fact, the removal of water from sericin during freeze–drying led to aggregation and the formation of less soluble β–sheets [51]. Several authors have proposed the use of lyoprotectants, such as mono–, di– and polysaccharides, to improve the protein–drying process. These molecules, in fact, can replace the water and establish new hydrogen bonds with sericin, thus sustaining its structure during lyophilization [8]. As expected, the sericin that was freeze–dried with trehalose (fd_ST) presented a good cake–like appearance with little shrinkage and collapse. Moreover, this powder showed slightly better handling properties than the protein alone. The fd_SM appeared as a cake similar to the product of the more conventional use of T as a lyoprotectant, demonstrating that methylated–β–CD can also interact with sericin by creating a shield between the protein hydrophobic surfaces, thus preventing their interaction and aggregation in solution. Indeed, it is known that CDs are able to include hydrophobic amino acids inside their cavities and provide stabilizing ability during drying [32]. Analogous results were obtained with hydroxypropyl–β–CD, which was found to be effective in stabilizing proteins during the freeze–drying of LDH protein at a concentration below 1.0% *w*/*w* [52].

Nevertheless, despite their more uniform flat surface and voluminous cake–like appearance, compared to fd_S, the products containing carrier agents (fd_ST and fd_SM) were also clearly electrostatic. In addition, all the powders obtained via freeze–drying presented a glassy and fragile appearance, thus explaining their poor handling properties. The spray–dried powders, on the other hand, were made up of white and nearly spherical microparticles, and generally seemed to be easier to handle and less electrostatic than the freeze–dried ones.

**Figure 1:**
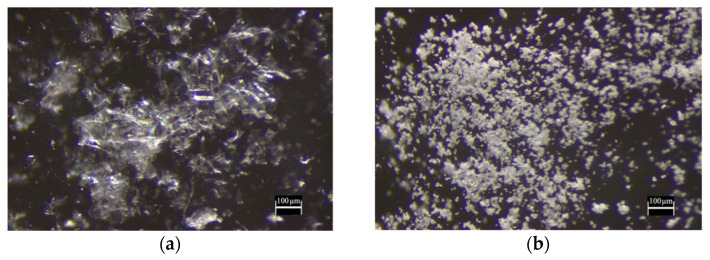
Stereomicroscope images of (**a**) fd_SM and (**b**) sd_SMT. Magnification 100×.

The studied powders were inspected by stereomicroscope and SEM. As an example, the stereomicroscope images of fd_SM and sd_SMT are shown in Figure 1.

**Figure 2:**
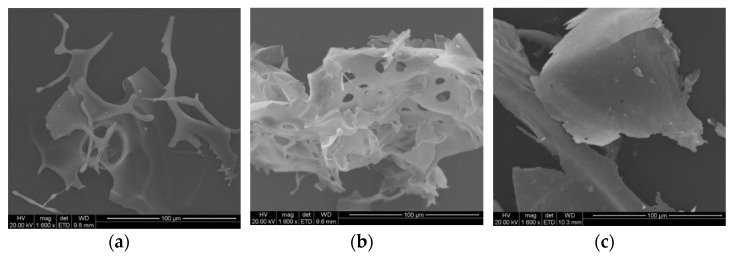
SEM images of (**a**) fd_S, (**b**) fd_ST and (**c**) fd_SM. Magnification 1600×.

Figure 2a (fd_S) displays globular and a more fibrillar structure that is characteristic of sericin [51] while the SEM images of the fd_ST and fd_SM powders show large laminar flakes (Figure 2b,c) whose aspect is irregular and discontinuous. In addition, the presence of small holes in the fd_ST particles (Figure 2b) may indicate an initial collapse of the cake, thus explaining the poor handling characteristics of this freeze–dried powder. However, all the freeze–dried particles appear visibly friable. These peculiar features negatively affected the handling characteristics of the fd_powders and made them unsuitable for fruitful manufacturing processes.

**Figure 3:**
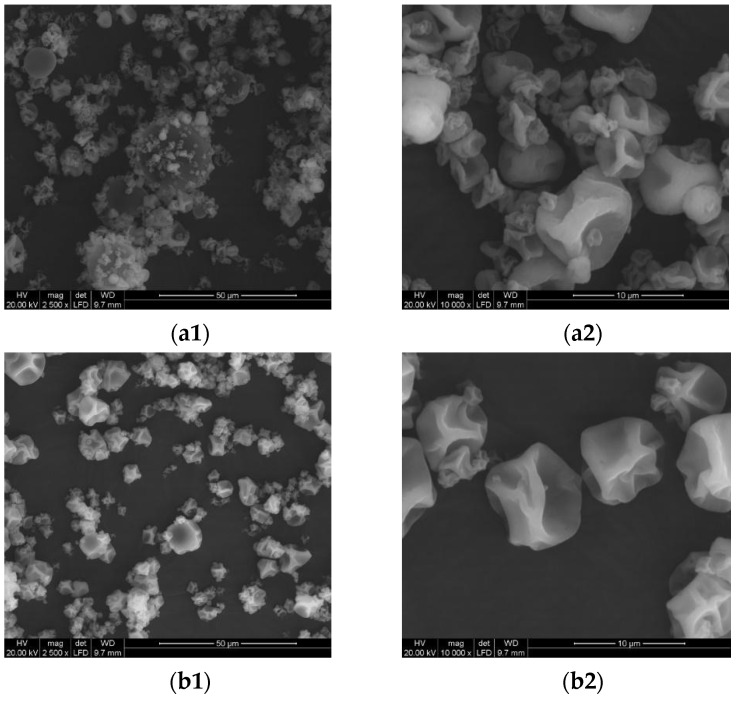
SEM images of (**a**) sd_S and (**b**) sd_SMT. Magnification (**1**) 2500×; (**2**) 10,000×.

The sd_S and sd_SMT powders were made up of microparticles characterized by rough surfaces, with hollows and wrinkles due to the partial collapse of the particles [53]. In particular, the sd_SMT powder (Figure 3b) presented a lower grade of aggregation compared to the spray–dried protein (Figure 3a) and a better homogeneity in size.

#### 2.2.2. Percentage Yield of Spray–Drying

The percentage yields of all the sd_powders are reported in Table 1 as mean value and standard deviation (*n* = 3). The carriers positively affected protein drying: this may be ascribed to the low molecular weight of the carrier agents, meaning that these small molecules can penetrate the sericin structure, stabilizing the protein during the spray–drying and so preventing its aggregation. In particular, the process yield increased from 34.4 ± 5.2% *w*/*w* of sd_S to 43.8 ± 4.9% *w*/*w* of sd_ST. The combination of T and M carriers improved the process yield compared to the spray–dried carrier alone (sd_MT: 49.6 ± 1.9% *w*/*w* vs sd_M: 23.6 ± 5.3% *w*/*w* and sd_T: 36.9 ± 2.7% *w*/*w*). The spray–drying of the sericin dispersion containing T and M gave a process yield that was even higher (sd_SMT: 58.9 ± 1.2% *w*/*w*), at more than 50% *w*/*w*, which is the landmark value beyond which the process can be defined as successfully performed [54]. This satisfying yield, which is noticeably larger than that of sericin alone and fairly good for laboratory–scale production, has to be considered even more significant in view of the small amount of dispersion (50 mL) used in all of the drying treatments. The increase in process yield may be ascribed to the improvement of the powder–handling characteristics, such as the particles having a lower tendency to disperse in the air and be electrostatic. These properties were observed during the powder recovery phase as well as at the end of the drying process. T and M carriers together may therefore have synergistic effect in inhibiting aggregation during spray–drying. This effect has also been reported for trehalose and another CD, hydroxypropyl–β–CD and for trehalose and bovine serum albumin (BSA) in the stabilization of monoclonal antibodies and xanthine oxidase, respectively [55,56].

The sericin that was dried without T and M carriers (sd_S) gave a powder that was made up of particles whose diameter was smaller than that obtained using the carrier agents (sd_SMT), as assessed by the PSD studies (see below). This characteristic may explain the difficulty in gathering the dried sd_S and the lower process yield obtained with this product.

A Kjeldahl analysis was performed on the sd_SMT to check the efficiency of the spray–drying process, together with the homogeneity of the dried powders. The obtained protein content was 22.6 ± 0.5% *w*/*w* (*n* = 3), which is in agreement with the theoretical amount of sericin in cocoons [16,49]. Therefore, besides confirming the high efficiency of the extraction technique, the Kjeldahl analysis also highlights that the loss of protein during the process was negligible.

#### 2.2.3. Thermal, Spectroscopic and Granulometric Analyses

**Figure 4:**
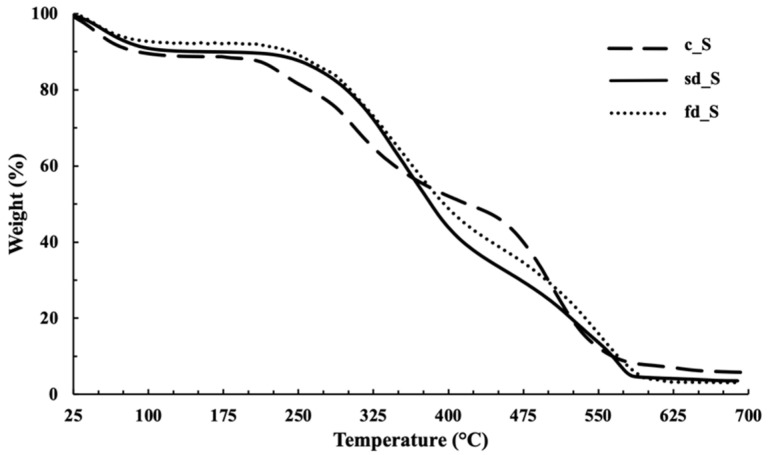
Thermogravimetric curves of the sericin powders.

**Figure 5:**
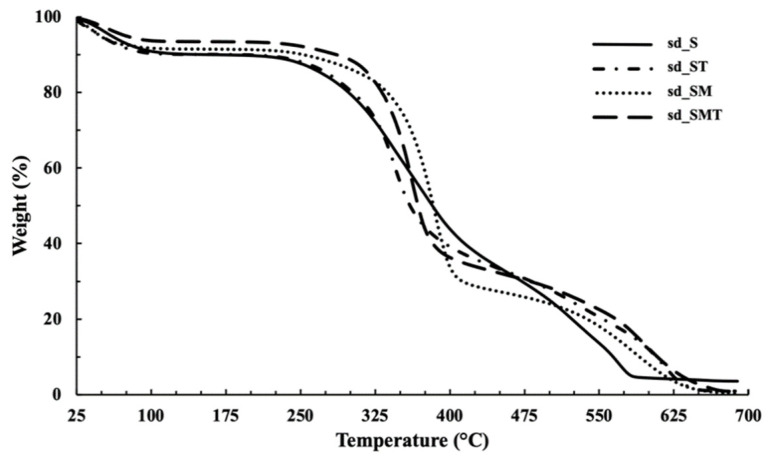
Thermogravimetric curves of all the spray–dried sericin powders.

Thermogravimetric analysis was used to determine the residual water content and investigate the decomposition behavior of the dried powders (Table 1, Figure 4 and Figure 5).

Firstly, a comparison between the various sericin powders, c_S, fd_S and sd_S was carried out (Figure 4). The corresponding TGA profiles present a first weight loss at between 25 and 125 °C, which is related to the residual moisture (between 8 and 10% approximately), according to the literature [16,23].

The well–separated steps between 200 and 680 °C (Figure 4) are related to protein degradation, consisting of deamination, decarboxylation and depolymerization, with the concomitant carbonization of the primary structure starting at 450 °C [16]. The comparison between the different sericin powders highlights a slight improvement in the thermal stability of the protein (c_S < fd_S < sd_S): an increase in the onset temperature of weight loss can be observed at 210 °C, 220 °C and 240 °C, respectively.

As expected, it can be seen that all of the powders were characterized by lower moisture content when the carrier agents were present. Indeed, the water percentages ranged between 4.5% and 5.5% for fd_SM and fd_ST powders, and between 6.1% and 8.7% for the spray–dried ones (sd_ST, sd_SM and sd_SMT). A good reduction in water content was detected, compared to sd_S, especially when the two carriers were used in combination (Table 1). These results can be attributed to the replacement of water molecules and the interactions between the components during the drying process.

A comparison of the thermal behavior of sd_S and sericin that was spray–dried in the presence of trehalose (sd_ST), methyl–β–cyclodextrin (sd_SM) or both (sd_SMT) is shown in Figure 5. The efficiency of the carriers is proven; the presence of T and M (sd_SMT) gave rise to the lowest moisture content of all the dried powders (Table 1). This result is clearly related to the highest spray–drying yield, which was around 59%.

The thermal profiles of the sericin powders that were freeze–dried in the presence of the T and M carriers (fd_ST and fd_SM) showed a degradation onset temperature of between 230 and 245 °C (data not reported), which is higher than that of fd_S, suggesting that both the stabilizing agents seem to be able to enhance the thermal stability of the protein.

Regarding the sd_powders (Figure 5), which are characterized by a residual water content that is higher than that of the fd_powders, an increase in thermal stability of the protein is negligible for sd_ST, while it is significant in the presence of the cyclodextrin (sd_SM and sd_SMT). These results may be explained by referring to the natural role of trehalose, which is produced by unicellular organisms to protect proteins from denaturation after exposure to stress [57], and to the ability of CDs to enhance the physical stabilization of proteins against different kinds of stress conditions, such as thermal stress [50].

Despite the higher efficacy of the freeze–drying process (Table 1) in drying and removing bonded water, this technique is more expensive and time–consuming than other drying processes. Considering the results discussed so far (i.e., greater thermal stability and better powder handling), only the sd_powders were characterized further.

**Figure 6:**
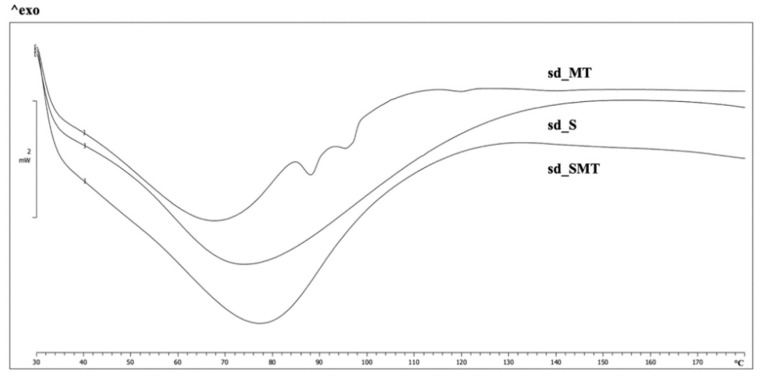
DSC curves of sd_MT, sd_S and sd_SMT powders.

The results of the DSC analyses (Figure 6) show a broad peak in the temperature range 40–100 °C, which is attributable to the evaporation of the residual water. Although the glass transition temperature (Tg) has not been determined by DSC, the curves of the powders containing sericin show at about 50 °C a signal which probably refers to the glass transition of the protein. This transition is more emphasized in the case of sd_S profile and it is consistent with the moisture content, which is higher than the sd_SMT one. The sd_MT curve displays two endothermic signals at about 90 and 95 °C, which are ascribable to the carriers’ bonded water. The absence of these two endothermic events in the sd_SMT powder profile may be explained by a strong interaction that occurs between sericin and the carriers. In fact, the stabilizer effect of T and M is due to the formation of hydrogen bonds with the protein. The original bounds existing between sericin and water molecules were broken, the carrier agents replaced water molecules which became bound–free and easier to remove.

CDs seem to be able to interact with proteins via hydrogen bonding, similarly to saccharides and polyols. In the case of M, it has also been hypothesized that the stabilization may occur during the freezing phase of lyophilization, and thanks to the surfactant–like effect of CDs during spray–drying atomization [32,39]. Finally, in accordance with a recent study, which demonstrated that the spray–drying process does not affect the stability of the sericin protein and its biological properties [23], no endothermic signals at 120–130 °C, which would be attributable to protein denaturation [16], are evident in the sericin–containing spray–dried powders (sd_S and sd_SMT).

**Figure 7:**
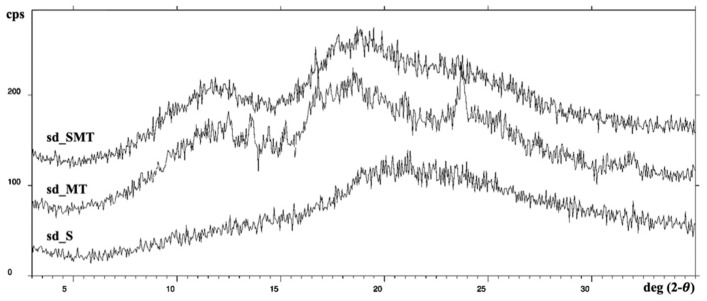
XRPD spectra of sd_S, sd_MT and sd_SMT powders.

The XRPD results, performed on the sd_powders, are also in agreement with those obtained from the DSC analysis; the spectra of sd_S and sd_SMT are similar and show a halo pattern, indicating the amorphous state of the powder components (Figure 7). This is not unexpected, as the spray–drying technique is usually employed to produce non–crystalline solids. However, the mixture of the carrier agents, which was processed via spray–drying (sd_MT) under the same conditions as the sericin–containing powders, shows some sharp diffraction peaks due to the molecules of water linked to M and T. Therefore, according to the DSC results, the XRPD spectra highlight the filling capability and the removal of the bound water of both trehalose and methyl–β–cyclodextrin toward the protein, and their interaction. The carriers probably replaced the protein water molecules and lost their bound water during the drying process in the presence of sericin.

The granulometric analysis (PSD) revealed that sd_S and sd_SMT were made up of particles of different sizes; the sd_S powder was characterized by a d(0.9) 3.616 µm and a d(0.1) 0.973 µm, whereas the sd_SMT powder particles were greater in diameter, with values of d(0.9) 7.169 µm and d(0.1) 2.045 µm (data are reported as average particle diameters). The increase in particle dimensions when the disaccharide (T) and the CD (M) were present confirms the better handling of the sd_SMT powder and strengthens the notions of the interaction between the protein and the carrier agents. The aforementioned values, combined with the volume weighted mean [D4,3] values of the sd_S (2.161 µm) and sd_SMT (4.289 µm) powders, suggest that the carrier agents were able to reduce the collapse of the protein during the drying process, leading to particles with higher volume. The width of the distribution was evaluated using the span calculation. The similar span values of sd_S and sd_SMT (1.339 and 1.334, respectively) indicate good particle homogeneity and comparable distribution despite their difference in size.

**Figure 8:**
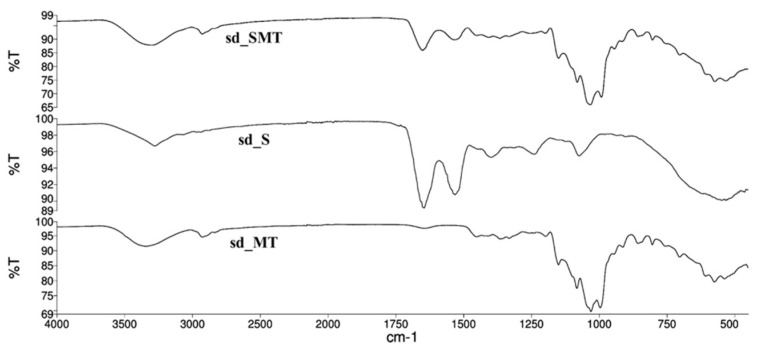
ATR–FTIR spectra of sd_SMT, sd_S and sd_MT powders.

The ATR spectra of the sd_S, sd_MT and sd_SMT powders are reported in Figure 8. The characteristic bands of sericin, related to amide I, amide II and amide III, at 1630–1650, 1520–1540 and 1230–1270 cm^−1^ respectively [16], are still evident in both sd_S and sd_SMT, confirming that the spray–drying process did not alter the protein structure.

#### 2.2.4. Microbiological Analysis

As expected, the total microbial count of the sericin dispersion, obtained from the degumming process in an autoclave, was low: 3.33 ± 0.58 CFU/mL, and fungi were absent.

For the sd_SMT powder, the total microbial count was 1.00 ± 0.16 × 10^3^ CFU/g, while the fungal count was 1.00 ± 0.11 × 10^2^ CFU/g. These values fall within the microbiological limits for Category–2 cosmetic products (not specifically intended for children under 3 years, eye areas and mucous membranes), as reported in the EU’s SCCS (Scientific Committee on Consumer Safety) notes of guidance [58].

## 3. Materials and Methods

### 3.1. Materials

Silk sericin (S) was extracted from the cocoons of *Bombyx mori* that were mechanically isolated from the silkworms, which were kindly supplied by a local small–scale experimental breeding center. Trehalose dihydrate (T), methyl–β–cyclodextrin (M) and commercial sericin (c_S) were purchased from Merck Life Science (Milan, Italy). All other materials were of analytical grade and used as received.

### 3.2. Methods

#### 3.2.1. Sericin Extraction

Sericin was obtained using a high–temperature and high–pressure process, which is one of the most widely utilized methods for the removal of sericin from cocoons [47]. The degumming process was thus conducted in an autoclave (Steristeam Labclave, Turin, Italy), at 120 °C for 15 min at 2 bar pressure, in 40 mL purified water/g cocoon [27,49]. The dispersion obtained after the separation from the degummed fibroin fibers was stored at 4 °C. The extraction process has been performed in triplicate.

#### 3.2.2. Freeze–Drying Process

After the degumming process, the original sericin dispersion and those also containing 1% *w*/*v* of T or M were freeze–dried (Epsilon 2–6D LSCplus freeze dryer, Martin Christ, Osterode am Harz, Germany). Each batch was constituted of n. 100 glass vials (capacity 5 mL) filled with dispersion volume of 2 mL.

In addition, lyophilization was used not only to obtain sericin powder, but also to evaluate the extraction efficiency of the degumming process: the recovery of protein was calculated via in bulk freeze–drying of known volumes of dispersions.

Both the processes, in vials and in bulk, were conducted in triplicate.

The freeze–drying parameters were taken from the literature [59] and appropriately modified: the freeze drier was operated at −35 °C and the samples, after being transferred into vials, were frozen at a controlled shelf temperature of −35 °C until the end of the freezing phase that lasted 120 min. The initial shelf temperature in the primary drying process was kept at −35 °C and 0.133 mbar for 20 min. The samples were then heated to −20 °C at 0.2 °C/min and kept under these conditions for 20 h. In the secondary drying process, the shelf temperature was increased by 0.2 °C/min to 4 °C and maintained until the process was completed, i.e., for 4 h at 0.133 mbar. The aforementioned parameters were kept constant for the drying of all the dispersions in order to attribute a possible different final moisture content to the interaction between sericin and M or T, rather than to the different process conditions.

To monitor the process (freezing, primary– and secondary–drying steps), two sensors were used, both previously placed into the vials containing the sericin dispersions. In particular, PT100 thermoresistance (Tersid S.r.l., Milan, Italy) was used to detect the temperature, while LyoRx sensor (Martin Christ) to monitor the electrical resistance and verify that the samples were completely frozen within the set conditions. Moreover, to reduce the risk of aggregation during the drying process, the dispersions were diluted 1:2 with purified water. The obtained freeze–dried powders (fd_powders) were stored in hermetically sealed vials.

#### 3.2.3. Spray–Drying Process

The original degummed sericin dispersion, those containing 1% *w*/*v* T or M, and the 1:1 mixture of both the carrier agents (2% *w*/*v*) were converted into powders using a laboratory spray dryer (Mini Spray Dryer B–290, Büchi, Flawil, Switzerland). To evaluate their processability in this drying equipment, the dispersions were subjected to viscosity determination before the treatment using a Brookfield DV–II+ viscometer (Brookfield, Middleboro, MA, USA) with the spindle 18, at shear rate 26.4 s^−1^ and 25 °C.

The dispersions, kept under stirring at room temperature, were fed into the spray dryer by a peristaltic pump at a feed rate of 6 mL/min. They were then atomized using a two–fluid nozzle atomizer 0.7 mm, combined with a 1.4 mm cap, at a gas flow of 601 L/min. The aspirator rate was set at its maximum gas flow rate (35 m^3^/h) and the relative humidity condition was kept constant using a dehumidifier. The inlet temperature was set at 120 °C. A glass fiber was selected as the outlet filter of the spray dryer. With these drying–process parameters, the resulting outlet temperature was 75.3 ± 0.9 °C, which avoids the protein denaturation that usually occurs at temperatures above 80 °C [60].

The obtained powders (sd_powders) were stored in hermetically sealed vials. The percentage yield (% *w*/*w*) of the spray–drying process was calculated as the ratio between the weight of the powder recovered after the spray–drying treatment and the solid present in the dispersions containing sericin alone (calculated via freeze–drying, see paragraph 2.2.2), without and with the carrier agents.

The abbreviations used in the manuscript are reported in the following Table 2.

#### 3.2.4. Physico–Chemical Characterization of Sericin Powders

The residual water content and the thermal decomposition behavior of all the freeze–dried and spray–dried powders were evaluated using thermogravimetric analysis (TGA). The samples (approximately 2–5 mg) were heated at a scan rate of 10 °C/min, under a 20 mL/min nitrogen purge from 25–700 °C by a Pyris™ 1 thermogravimetric analyzer (Perkin Elmer, Milan, Italy). Likewise, the commercial sericin (c_S) and the single components (T, M), which were processed using the same drying techniques as the sericin dispersions, were analyzed with TGA. The instrument was previously calibrated with reference standards: alumel, nickel, perkalloy and iron, and measurements were carried out at least in triplicate.

The surface and morphology of all the dried sericin powders were observed both with a Leica S9i stereomicroscope (Leica, Wetzlar, Germany) and a Quanta™ 200 scanning electron microscope (SEM) (FEI, Eindhoven, The Netherlands) at 20 kV. Samples were coated with a 40 nm golden film, prior to SEM analysis.

The sd_powders were also subjected to differential scanning calorimetry (DSC), granulometric investigation by particle size distribution (PSD), X–ray powder diffraction (XRPD) and attenuated total reflectance–Fourier transform infrared (ATR–FTIR) spectroscopy.

DSC was conducted on accurately weighed samples (2–3 mg) of the spray–dried sericin alone (sd_S), sericin spray–dried with M and T (sd_SMT) and a spray–dried mixture of trehalose and cyclodextrin without sericin (sd_MT), using a DSC 1 Stare System (Mettler–Toledo, Eindhoven, Italy). The analyses were performed in aluminum crucibles, at 10 °C/min and in a 30–170 °C temperature range under an 80 mL/min nitrogen purge. Indium was utilized for calibration.

For the particle–size assessment, the powders were dispersed in ethanol and the resulting suspensions were sonicated for 5 min and then analyzed. The laser light scattering granulometer (Mastersizer 2000, Malvern Instruments, Malvern, United Kingdom) was set at a 1.36 refraction index, and five replicates (of 10 s each) were performed for each sample. The acquired data were used to find the span value of the powders.

The XRPD of the sd products was carried out on the sd_powders of sericin alone, those with the carrier agents, to observe their possible structural differences, and on the physical mixture of M and T by an APD 2000 diffractometer (G.N.R., Novara, Italy) in the scan range 3–35° 2θ and at a scan speed of 0.04° 2θ.

The ATR–FTIR analysis of sd_S, sd_MT and sd_SMT were carried out using a Spectrum Two™ IR spectrometer (Perkin Elmer). The spectra were recorded between 4000 and 450 cm^−1^ (4 cm^−1^ resolution, 16 scans).

Moreover, the Kjeldahl analysis was performed to estimate the nitrogen content of the sd_SMT powder. A weighed amount of powder was processed at 420 °C for 1 h with a tablet of catalyst (1.5 g K_2_SO_4_–7.5 mg Se), 98% *w*/*w* H_2_SO_4_ (8 mL) and 30% *w*/*w* H_2_O_2_ (2 mL). The distillation was carried out after the addition of purified water (30 mL) and 40% *w*/*w* NaOH (40 mL) in a Kjeltec™ 8100 Tecator Line (Foss, Hilleroed, Denmark). The excess of H_2_SO_4_ (0.1 N), which had been added to the distillate of each sample beforehand, was detected via titration with an NaOH solution (0.1 N). The conversion factor of 6.25 was introduced to transform the grams of nitrogen into grams of protein. The results are reported as mean value and standard deviation (*n* = 3).

##### 3.2.5. Microbial Counts

Microbial counts were carried out in triplicate on the sericin dispersions, after the separation of the degummed fibroin fibers, and on the sd_SMT powder by means of the viable cell counting method. Tryptic soy agar (TSA) was used for bacterial and fungal counts (total count), while Sabouraud dextrose agar, with added chloramphenicol (SDA + CFA), was used for the fungal counts. After incubation at 30 °C for five days, the colonies were counted and the results are expressed as colony forming units per milliliter (CFU/mL) for sericin dispersions, and as colony forming units per gram (CFU/g) for the sd_SMT powder.

## 4. Conclusions

Trehalose and methyl–β–cyclodextrin, which have been used in this study to prepare sericin powders via the freeze–drying and spray–drying techniques, can be recommended as effective carrier and carrier agents for this protein.

Indeed, these two molecules have been demonstrated to improve the protein–drying process. In particular, compared to the sericin powder (sd_S), the protein dried in the presence of both the carriers (sd_SMT) gave the lowest moisture content (9.8 ± 0.3% *w*/*w* vs. 6.1 ± 0.2% *w*/*w*). This result can be ascribed to the replacement of water molecules and to the interactions between the components during the drying process. Moreover, sd_SMT resulted in the highest process yield (58.9 ± 1.2% *w*/*w* vs. 34.4 ± 5.2 *w*/*w* of sericin alone) and the best handling characteristics, especially for a laboratory–scale process. This powder is currently under deeper evaluation (i.e., structure and biological activity) as functional ingredient candidate in rinse–off hair–care preparations, such as masks and conditioners.

Furthermore, in view of the scale–up of the manufacturing of sericin–containing powders, a DoE approach will be used to optimize the spray–drying of the protein dispersion, not only using different carriers, but also cationic stabilizing agents.

## Figures and Tables

**Table 1:** List of abbreviations used in the text.

Samples	fd_Powders	sd_Powders	
	Moisture (% *w*/*w*)	Moisture (% *w*/*w*)	Yield (% *w*/*w*)
S	7.9 ± 0.1	9.8 ± 0.3	34.4 ± 5.2
T	5.2 ± 0.1	5.2 ± 0.3	36.9 ± 2.7
M	2.2 ± 0.2	5.0 ± 0.3	23.6 ± 5.3
MT	---	8.2 ± 0.1	49.6 ± 1.9
ST	5.5 ± 0.3	8.7 ± 0.1	43.8 ± 4.9
SM	4.5 ± 0.6	8.1 ± 0.1	38.0 ± 5.3
SMT	---	6.1 ± 0.2	58.9 ± 1.2

Note: moisture content of c_S = 8.6 ± 3.5%.

**Table 2:** Percentage moisture content in fd_ and sd_powders and spray–drying process yields. Data are reported as mean value and standard deviation (*n* = 3).

Materials	Freeze–Dried Powders	Spray–Dried Powders
Silk sericin (S)	fd_S	sd_S
Trehalose dihydrate (T)	fd_T	sd_T
Methyl–β–cyclodextrin (M)	fd_M	sd_M
Methyl–β–cyclodextrin + Trehalose dihydrate (MT)	---	sd_MT
Silk sericin + Trehalose dihydrate (ST)	fd_ST	sd_ST
Silk sericin + Methyl–β–cyclodextrin (SM)	fd_SM	sd_SM
Silk sericin + Methyl–β–cyclodextrin + Trehalose dihydrate (SMT)	---	sd_SMT

## Data Availability

Data is contained within the article or supplementary material.
